# Information Wanted

**Published:** 1879-04

**Authors:** 


INFORMATION WANTED.—The following is an exact copy of a writing that we saw recently on a doctor's slate: "Miss Jones said can his daughter Martha eat chicken soup out on First St. between federal and Coutts St."—*So. Clinic.*

